# Age-related changes in rat bone-marrow mesenchymal stem cell plasticity

**DOI:** 10.1186/1471-2121-12-44

**Published:** 2011-10-12

**Authors:** Faizal Z Asumda, P Bryant Chase

**Affiliations:** 1 Institute of Molecular Biophysics, Florida State University, Tallahassee, USA; 2 Department of Biological Science, Florida State University, Tallahassee, USA

## Abstract

**Background:**

The efficacy of adult stem cells is known to be compromised as a function of age. This therefore raises questions about the effectiveness of autologous cell therapy in elderly patients.

**Results:**

We demonstrated that the expression profile of stemness markers was altered in BM-MSCs derived from old rats. BM-MSCs from young rats (4 months) expressed Oct-4, Sox-2 and NANOG, but we failed to detect Sox-2 and NANOG in BM-MSCs from older animals (15 months). Chondrogenic, osteogenic and adipogenic potential is compromised in old BM-MSCs. Stimulation with a cocktail mixture of bone morphogenetic protein (BMP-2), fibroblast growth factor (FGF-2) and insulin-like growth factor (IGF-1) induced cardiomyogenesis in young BM-MSCs but not old BM-MSCs. Significant differences in the expression of gap junction protein connexin-43 were observed between young and old BM-MSCs. Young and old BM-MSCs fused with neonatal ventricular cardiomyocytes in co-culture and expressed key cardiac transcription factors and structural proteins. Cells from old animals expressed significantly lower levels of VEGF, IGF, EGF, and G-CSF. Significantly higher levels of DNA double strand break marker γ-H2AX and diminished levels of telomerase activity were observed in old BM-MSCs.

**Conclusion:**

The results suggest age related differences in the differentiation capacity of BM-MSCs. These changes may affect the efficacy of BM-MSCs for use in stem cell therapy.

## Background

Age-related changes in adult stem cells contribute to the decline in tissue regenerative capacity [1,2]. Adult stem cells are the primary driving force for tissue, and hence organ specific self-renewal. The observed reduction in tissue regenerative capacity suggests diminished stem cell numbers in addition to compromised differentiation and specific lineage commitment ability [2,3]. In rats and mice, aging compromises the efficacy of MSCs geared towards regeneration of damaged myocardial tissue [4,5]. The aging micro-environment has also been shown to pose an inhibitory effect on adult stem cell mediated regeneration [3,6]. Genes involved in stemness, genomic integrity and regulation of transcription are age-repressed in MSCs undergoing replicative senescence *in vitro *[7,8]. In this study, MSCs derived from the bone marrow of 4 month old rats (young) were compared to those derived from 15 month old rats (old).

## Results

### Characterization of young and old BM-MSCs and assessment of changes in pluripotent marker expression

**Figure 1:**
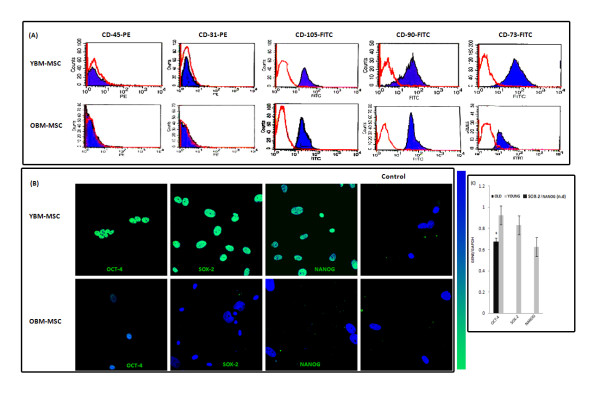
**Assessment of pluripotent marker expression and characterization of young and old BM-MSCs:** (A) Passage 3 BM-MSCs from young and old rats were harvested and labeled with a PE-coupled antibody against CD45 and CD31, and FITC-coupled antibodies against CD105, CD90 and CD73 or immunoglobin isotype control IgGs and analyzed with FACS. Histograms show counts for specific antibody staining profiles (blue). (B) Immunocytochemical analysis demonstrates that old BM-MSCs express the pluripotent maker Oct-4 but fail to express Sox-2 and NANOG. (C) qPCR analysis for the relative expression of Oct-4, Sox-2 and NANOG in young and old BM-MSCs. We failed to detect Sox-2 and NANOG in old BM-MSCs. Columns show the combined mean ΔΔC_q _values of young (n = 4) and old rats (n = 4) for each marker. Data represent relative expression of transcripts normalized relative to GAPDH and expressed as the Mean ± SEM for three biologically independent experiments (n = 3). The '‡' sign indicates a significant difference between the indicated group and any passage 3 groups from younger donors (*p <*0.05). (n.d ~ not detectable)

The yield of MSCs from the bone marrow of all four individual old rats was lower than that of young rats. We observed higher numbers of plastic adherent colonies in all four individual young rat cultures 24 hours after initial seeding of equal volumes (15 mL) of bone marrow aspirates from each individual animal. Young rat cultures expanded rapidly, and reached over 80% confluence before day 5 post primary isolation. In comparison, adherent colonies only became apparent in all four old rat cultures after 6 days post primary isolation (Additional file 1). Young BM-MSCs displayed elongated fibroblast-like spindle shaped morphology (Additional file 1). Old BM-MSCs displayed a spread out, flat enlarged morphology (Additional file 1) with nuclei that appeared larger in size. Using a Cedex HiRes non-flow imaging cytometer, we observed larger average cell diameter in trypsinized old BM-MSC cultures (Additional file 1). All young and old BM-MSC cultures were negative for CD45 and CD31, and positive for CD105, CD90 and CD73 (Figure 1A). We tested the hypothesis that there might be age-related differences in the expression profile of Oct-4, Sox-2 and NANOG in BM-MSCs derived from young and old bone marrow. While old BM-MSCs expressed Oct-4, the fluorescence signal intensity was significantly lower in comparison to that observed in young MSC cultures (Figure 1B). The immunocytochemical observations were corroborated by qPCR; we failed to detect Sox-2 and NANOG in old BM-MSCs but all three markers were detected in young BM-MSCs by qPCR (Figure 1C).

### Multilineage differentiation capacity is diminished in BM-MSCs from old rats

**Figure 2:**
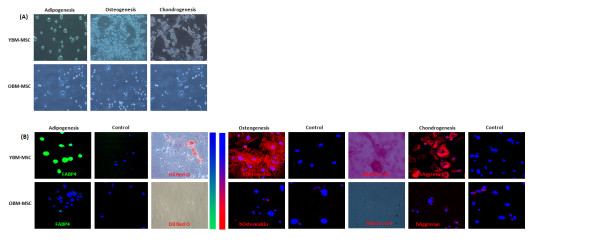
***In vitro *differentiation capacity of young and old BM-MSCs: (A) Representative micrographs from each experimental group showing morphological change after 21 days of differentiation**. (B) Results of adipogenic, osteogenic and chondrogenic differentiation. Representative micrographs showing anti-FABP4, Oil Red O, anti-osteocalcin, Alizarin Red-S and anti-aggrecan staining in cultured BM-MSCs from each experimental group.

We assessed the plasticity of young and old BM-MSCs by inducing differentiation into fat forming adipocytes, bone forming osteocytes, cartilage forming chondrocytes and cardiomyogenic cells. We observed the accumulation of triglycerides in the cytoplasm of young BM-MSCs after 48 hours of exposure to adipogenic differentiation media. Young BM-MSCs progressively changed morphology, and the size and number of lipid vacuoles formed increased over 8 days of differentiation. Transformation peaked at 8 days of differentiation, after which all young BM-MSCs in each culture plate became fully formed adipocytes and detached from the culture surface into suspension (Figure 2A). The resulting adipocytes stained positive for oil red-O and the adipocyte lipid-binding protein FABP4 (Figure 2B). Old BM-MSC cultures changed morphology but failed to differentiate over 21 days of induction in adipogenic, osteogenic and chondrogenic differentiation media (Figure 2A). We did not detect FABP4 in old BM-MSC cultures and oil red-O staining was negative (Figure 2B). We also observed the production of a mineralized matrix and the formation of bone nodules *in vitro *in young BM-MSC cultures after 48 hours of exposure to osteogenic differentiation media. Osteogenic differentiation of young MSC cultures peaked at day 8 with a complete change in cell morphology and gradual detachment of bone nodules into suspension (Figure 2A). Young MSC cultures stained positive for anti-osteocalcin and alizarin-red (Figure 2B). After 8 days of exposure to chondrogenic differentiation media, we observed the formation of proteoglycan-rich soft collagen matrix in young BM-MSC cultures (Figure 2A). Transformation peaked at day 8 with a complete change in BM-MSC morphology and detachment of fully formed chondrocytes into suspension (Figure 2A). The differentiated chondrogenic cells also stained positive for anti-aggrecan (Figure 2B). Fluorescence signal intensity as detected by immunocytochemistry was significantly higher in young BM-MSC cultures (Additional file 2).

**Figure 3:**
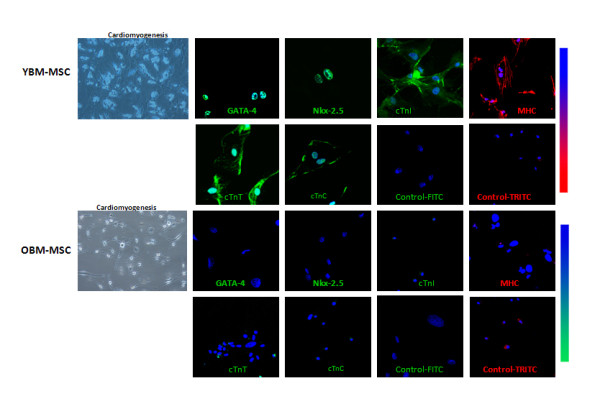
**Assessment of cardiac specific lineage commitment potential in young and old BM-MSCs by Immunocytochemistry: Results of cardiomyogenic differentiation after 21 days**. Representative micrographs showing anti-GATA 4, Nkx2.5, cTnI, cTnT, cTnC and MHC staining in cultured BM-MSCs from each experimental group.

To further assess self-renewal and differentiation capacity in young and old BM-MSCs, we induced cardiomyogenesis with a cocktail mixture of bone morphogenetic protein (BMP-2), fibroblast growth factor (FGF-2) and insulin-like growth factor (IGF-1). Old BM-MSCs progressively changed morphology and in some cultures, we observed cell death in response to cardiomyogenic differentiation media (Figure 3). Young BM-MSC cultures first proliferated to become over confluent and progressively changed morphology. We observed progressive enlargement and elongation in young BM-MSC cultures with the formation of ball and stick-like morphology (Figure 3). By day 10 of induction, the differentiating BM-MSCs formed a long cytoplasmic process with a vacuolated appearance (Figure 3) similar to that observed in adipogenesis but with uniquely different surface and morphological characteristics [9,10]. We did not observe contracting cells typical of NVCM. However, differentiating cells connected to adjoining cells, and formed myotube-like structures and string-bead like formations (Figure 3). Immunostaining of differentiated cells for GATA 4, Nkx2.5, cTnI, cTnT, cTnC and myosin heavy chain was strongly positive and revealed bi-nucleation (Figure 3 and Additional file 2). Induced old BM-MSCs failed to express the same set of cardiac markers (Figure 3 and Additional file 2). The immunocytochemical observations were corroborated by qPCR; we failed to detect significant levels of Nkx2.5, GATA 4, cTnI, cTnT, cTnC and cTropomoysin transcripts in old BM-MSCs (Additional file 3A).

### Young and old rat BM-MSCs form gap junctions and undergo cardiomyogenesis in co-culture with NVCM

**Figure 4:**
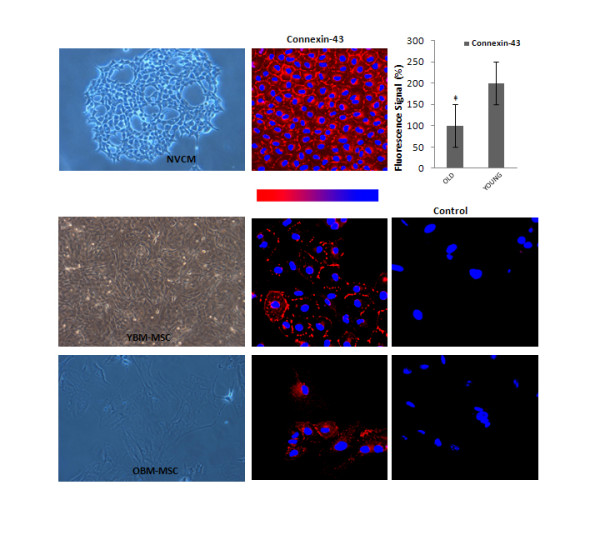
**Immunocytochemical analysis of Connexin-43 staining: Representative images of anti-connexin-43 staining in cultured BM-MSCs from each experimental group**. Columns show fluorescence signal intensity based on three independent differentiation experiments (n = 3) per animal. The '‡' sign indicates a significant difference between the indicated group and any passage 3 groups from younger donors (*p <*0.05). Error bars designate means ± SEM.

**Figure 5:**
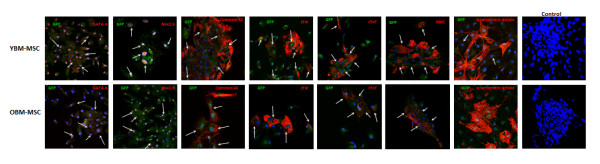
**Immunocytochemical analysis of Cardiomyogenic differentiation of BM-MSCs co-cultured with NVCM: Results of co-culture with NVCM after 14 days**. Representative micrographs of anti-GATA 4, Nkx2.5, connexin-43, cTnI, cTnT, MHC and α-sarcomeric actinin staining in cultured BM-MSCs from each experimental group.

The presence of cardiac connexins which form the basis for cell-to-cell communication has been shown in human and rat MSCs and between MSCs and cardiomyocytes [11,12]. We hypothesized here that there might be age-related differences in the expression of the gap junction protein connexin-43 in MSCs alone and in co-culture with ventricular cardiomyocytes. We observed the typical punctate staining pattern of connexin-43 in both young and old BM-MSC cultures and in NVCM (Figure 4). Fluorescence intensity of connexin-43 staining as detected by immunocytochemistry was significantly higher in young BM-MSC cultures (Figure 4). When layered over a subconfluent culture of NVCM, young and old BM-MSCs coupled and fused with adjacent NVCM colonies. Fused young and old BM-MSCs expressed GATA 4, Nkx2.5, Connexin-43, cTnI, cTnT and myosin heavy chain, but failed to express α-sarcomeric actinin in co-culture with NVCM (Figure 5).

### Assessment of DNA damage and telomerase activity in young and old BM-MSCs

**Figure 6:**
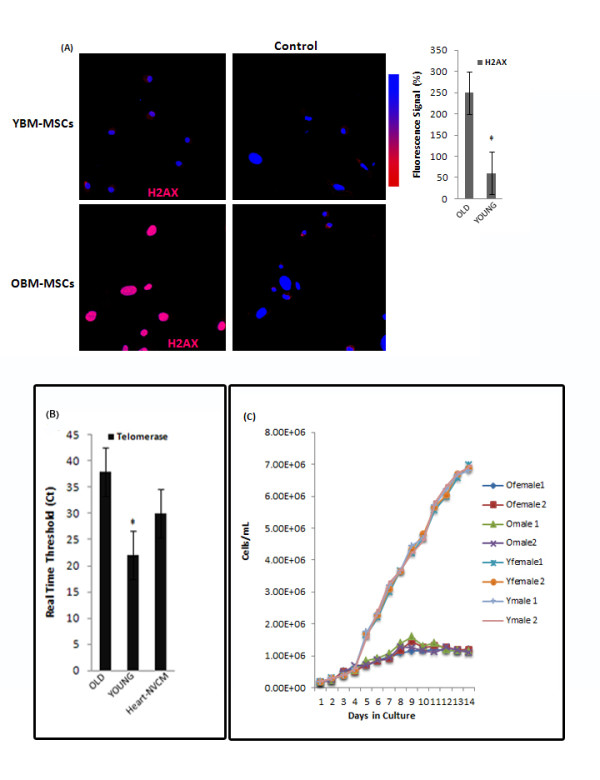
**Telomerase activity, growth and immunocytochemical assessment of DNA double strand break in young and old BM-MSCs: **(A) Representative micrographs of anti- γ-H2AX staining in cultured BM-MSCs from each experimental group. (B) Columns show comparison of telomerase activity between young and old BM-MSCs by qPCR. C_q _values for young BM-MSCs are significantly lower indicating higher enzymatically active telomerase. Data represent Mean ± SEM for three biologically independent experiments (n = 3). The '‡' sign indicates a significant difference between the indicated group and any passage 3 groups from older donors (*p <*0.05). (C) Passage 0 BM-MSCs from individual young and old rats were trypsinized, and enumerated with a Cedex HiRes non-flow imaging cytometer over 15 days.

The phosphorylated histone H2A variant, γ-H2AX is a sensitive indicator of DNA damage [13-16]. We postulated that MSCs derived from the bone marrow of old rats will express higher levels of γ-H2AX while displaying diminished levels of telomerase activity. We detected significantly higher levels of γ-H2AX, in old BM-MSC cultures (Figure 6A). We failed to detect expression of γ-H2AX in young BM-MSC cultures (Figure 6A). Furthermore, telomerase activity (Figure 6B) and population growth (Figure 6C) was significantly higher in young BM-MSC cultures. Transplanted MSCs have been shown to provide a major beneficial effect through paracrine signaling [17-20]; similar effects are to be expected when young and old BM-MSCs are transplanted. We therefore tested the hypothesis that young and old BM-MSCs might differentially express and secrete growth factors and cytokines. Old BM-MSCs expressed significantly lower levels of VEGF, EGF, IGF and G-CSF transcripts (Additional file 3B).

## Discussion

We demonstrated here using a rodent model that there were differences in BM-MSCs associated with the age of the animal from which the cells were isolated. We observed rapid expansion in young BM-MSC cultures and striking age-related differences in cell morphology, population size and proliferation. Young BM-MSCs displayed elongated fibroblast-like spindle shaped morphology. Old BM-MSCs displayed spread out, flat enlarged morphology which is consistent with late passage and extensively cultured BM-MSCs [7,21-23]. Consistent with age-related decreased proliferation, we observed diminished telomerase activity and significantly higher levels of the DNA double strand break marker γ-H2AX in old BM-MSCs. Human fetal bone marrow is known to contain approximately 1 in 10, 000 MSCs in comparison to 1 in 250, 000 in adults [24]. Consistent with our observations here, studies in rats, mice, and rhesus macaque monkeys indicate that there is a correlation between age and declining numbers of BM-MSC [25-27].

There is conflicting data with regard to the effect of aging on the bone forming ability of BM-MSCs [21,26,28,29]. A few studies find no effect, but the majority find age-related decline in differentiation potential [30]. In this study, old BM-MSCs failed to differentiate into osteocytes, chondrocytes and adipocytes. We assessed cardiomyogenic potential by stimulation with a cocktail mixture of BMP-2, FGF-2 and IGF-1, in co-culture with NVCM and with conditioned media derived from the co-culture system. Old BM-MSCs failed to commit to the cardiac lineage when induced with the cocktail mixture and conditioned media. We observed expression of the cardiac phenotype in both young and old BM-MSCs in co-culture with NVCM. When injected into infarcted myocardium, old rat BM-MSCs displayed scant myogenic transdifferentiation and failed to adopt host myocardial tissue architecture in comparison to young MSCs [4,5]. A potential drawback to co-culture is the fact that fusion of the two different cell types can be misinterpreted as transdifferentiation [31]. Expression of the cardiac phenotype in old BM-MSCs under co-culture conditions in this present study maybe explained in part by cell fusion.

We did not detect striations typical of NVCM in young and old BM-MSCs as evidenced by the lack of α-sarcomeric actinin staining in either cell type. Furthermore, a significant number of GFP positive cells in old BM-MSC cultures failed to express the cardiac proteins analyzed in this study. Positive staining for cardiac proteins was observed in regions of the co-culture system where GFP positive BM-MSCs had fused with NVCM. It is not clear that old BM-MSCs acquired a broader differentiation potential in co-culture with NVCM that enabled commitment to the cardiac lineage. Both young and old BM-MSCs expressed the gap junction protein connexin-43. Therefore, it is more probable that old BM-MSCs fused with NVCM and took on their characteristics. Great interest in BM-MSCs is based in part on their ability to improve function, angiogenesis and neovascularivation via paracrine and cytokine signaling; the same is to be expected of transplanted young and old BM-MSCs. The neovascularization and angiogenesis effects of BM-MSCs are enhanced by the presence of specific growth and cytokine factors [17,18,27]. In this study, we observed markedly lower levels of G-CSF, IGF, EGF and VEGF transcripts in old BM-MSCs.

The inability of old BM-MSCs to respond to differentiation media maybe explained in part by the fact that the BM-MSC isolation method typically results in slight heterogeneity in the cell population at early primary isolation due to hematopoietic stem cell contamination which affects the *in vitro *behavior of the cells. We observed higher contamination by hematopoietic stem cells in old BM-MSC cultures at early passage by flow cytometry (not shown). However, young and old BM-MSCs uniformly expressed CD90, CD73 and CD105, and failed to express CD45 and CD31 at passage 3. It is more likely that the related genes necessary for efficient adipogenesis, chondrogenesis, osteogenesis and cardiomyogenesis were age-repressed in our old BM-MSCs [27]. An Alternative explanation is that our old BM-MSCs had already partially differentiated to another lineage prior to induction with differentiation media. This is highly unlikely; the differentiation experiments in this study were carried out in triplicate (n = 3) on individual cell populations derived from the bone marrow of individual rats (n = 4) for each age group. We induced young and old BM-MSCs under uniform conditions at passage 3.

The uniform expression of CD90, CD105, CD73 and absence of CD31 and CD45 in either age group as observed in this study indicates homogeneity in the BM-MSC population. It does not necessarily correlate with multidifferentiation capacity. Hence, the complete loss of chondrogenic, osteogenic and cardiomyogenic potential may also be due to an age-related loss in multipotency. The combinatorial transcription factor interaction network of Oct-4, NANOG and Sox-2 which confers stemness and maintains pluripotency in embryonic stem cells is well documented [25,32,33]. Using an *in vitro *aging model, Yew et al. recently showed that BM-MSCs show decreased expression of Oct-4 and NANOG at late passage. They also showed that the knockdown of p21 rescues the loss in differentiation capacity and potentiates expression of Oct-4 and NANOG [7]. We observed age-related changes in the expression profile of the three pluripotency markers Oct-4, Sox-2 and NANOG. Old BM-MSCs expressed Oct-4, albeit at a lower intensity in comparison to young BM-MSCs, but failed to express Sox-2 and NANOG. While its presence does not automatically result in pluripotency, precise levels of Oct-4 are required to maintain pluripotency in embryonic stem cells, and its lack thereof results in germ line specific differentiation [34,35].

## Conclusion

Taken together, our observations here present definitive evidence that there are age-related changes in MSCs derived from the bone marrow of old rodents with regard to plasticity and basic stem cell biology. These results support the conclusion that the efficacy of BM-MSCs is compromised as a function of donor age.

## Methods

All experiments were performed in accordance with the guidelines of the Animal Care and Use Committee of the Florida State University and with the *Guide for the Care and Use of Laboratory Animals *(Department of Health and Human Services, publication No. NIH 86-23).

### Experimental design

This study investigated among other factors the effect of donor age on BM-MSC differentiation capacity into four mesodermal lineages. BM-MSCs were isolated from four individual "old" and "young" Sprague Dawley rats (15 months; 2 males and 2 females) and (4 months; 2 males and 2 females), respectively. We did not pool bone marrow aspirates from the individual animals; all experiments were performed on the separate cell lines isolated from each individual animal. MSCs are currently being used in several ongoing autologous clinical trials; hence the objective of such a study design was to provide a controlled analysis of the possible effect of individual donor age on molecular predictors of BM-MSC efficacy. Neonatal rats (age, 48 hrs, n = 12) were used for neonatal ventricular cardiomyocyte isolation.

### Antibodies

FITC- or phycoerythrin (PE) coupled antibodies against CD45 (559135; 1:100), CD31 (555027; 1:100), CD90 (554897; 1:100) and CD73 (551123; 1:100) were from (BD Pharmingen, San Diego, CA, USA). Mouse IgG1, k Isotype control (551954; 1:100) was from BD Pharmingen. Secondary FITC conjugated anti-goat (705-075-003; 1:200), anti-rabbit (711-095-152; 1:200), and TRITC conjugated anti-mouse (715-025-150; 1:200) antibodies were from (Jackson Immunoresearch, West Grove, PA, USA). Antibodies against CD105 (ab18278; 1:100), Ig2a Isotype control (ab18449; 1:100), cardiac troponin-I (ab19615-500; 1:100), cardiac Myosin Heavy Chain (ab15-100; 1:100) and alpha Sarcomeric actinin (ab9465-500; 1:100) were from (Abcam, Cambridge, MA, USA). Antibodies against p-histone γ-H2A.X (Ser-139) (sc-101696.; 1:200), SOX-2 (sc-17320; 1:100), Oct-3/4 (sc-9081; 1:100) and NANOG (sc-33760; 1:100) were from (Santa Cruz Biotechnology Inc., Santa Cruz, CA, USA). Antibody against connexin-43 (71-0700; 1:100) was from (Invitrogen, Carlsbad, CA, USA). Antibodies against osteocalcin (MAB1419; 1:100), aggrecan (AF1220), and FABP4 (AF1443; 1:100) were from (R&D Systems, Minneapolis, MN, USA).

### Bone marrow stem cell isolation

BM-MSCs were isolated from the femur and tibia of Sprague Dawley rats. Briefly, muscle and extra-ostial tissue were trimmed. Bone marrow plugs were flushed out, layered over 15 mL Ficoll solution (Sigma, St. Louis, MO, USA) and centrifuged at 1500 rpm. The cell layer at the interphase was aspirated, and washed with PBS (Fisher, Pittsburgh, PA, USA) in 1% FBS (Innovative Research, Novi, MI, USA). Bone marrow mononuclear cells were transferred to culture flasks with Dulbecco's modified Eagle's Medium-high glucose ([DMEM-HG] (Sigma) supplemented with 20% FBS (Innovative Research) at 37°C with 5% CO_2. _After 3 days of culture, the media and non-adherent cells were discarded and fresh media added at 3-4 day intervals. Seed culture cells were treated with 0.25% Trypsin-EDTA (Sigma) 7-14 days after the initial plating and labeled as passage 1.

### Neonatal heart cell isolation

Neonatal ventricular cardiomyocytes were isolated from 48 hours old Sprague Dawley rat litters (n = 12). Briefly, neonatal rats were anesthetized with halothane, decapitated, and hearts quickly removed and placed in 100 mm Petri dishes (Fisher) with cold phosphate-buffered saline solution (PBS; KCl, 2.7 mmol/L; NaCl, 136.9 mmol/L; KH_2_PO_4_, 1.5 mmol/L; Na_2_HPO_4_, 8.1 mmol/L [pH 7.3]). Hearts were minced into 1-mm^3 ^pieces, washed with PBS and digested in 0.1% trypsin, 0.3% collagenase and 0.5% DNAse (Worthington, Lakewood, NJ, USA) for 5 minutes at 37°C. The cell suspension was transferred into 30 mL of complete medium and centrifuged at 1500 rpm for 10 minutes. The cell pellet was resuspended in complete medium (F-12 nutrient mixture (Invitrogen) supplemented with 10% FBS and 10% horse serum (Invitrogen), plated in 60 mm dishes (Fisher) and cultured at 37°C in 5% CO_2_.

### BM-MSC morphology, population size and flow cytometry analysis

Primary isolation BM-MSCs derived from individual young and old rats were compared prior to the first passage. BM-MSCs were evaluated under phase contrast (BX61, Olympus; Tokyo, Japan) over 7 days beginning at 24 hours post primary isolation. At passage 3, we analyzed cells by flow cytometry; BM-MSCs from each individual animal were grown to confluency, trypsinized, and 1 × 10^6 ^cells were resuspended with phosphate-buffered saline (PBS) in FACS tubes (BD Bioscience). Cells were washed twice with cold PBS and incubated for 30 minutes on ice with the specific conjugated primary antibody, and corresponding isotype control. The immunostained cells were rinsed twice with cold PBS, and analyzed on a FACScan (BD Bioscience). To compare cell size and determine growth cycle, a Cedex HiRes non-flow imaging cytometer was used to enumerate BM-MSCs from each individual animal over 15 passages.

### *In vitro *differentiation

We tested the multilineage differentiation capacity of BM-MSCs by inducing differentiation into adipocytes, osteoblasts, chondrocytes and cardiomyogenic cells using the human stem cell functional identification kit (R&D Systems) and a combination of growth factors, according to the manufacturer's protocol. We induced adipogenesis, osteogenesis, chondrogensis and cardiomyogenesis over 21 day's culture in differentiation medium containing (hydrocortisone, isobutylmethylxanthine and indomethacin [R&D Systems]); (dexamethasone, ascorbate-phosphate, and β-glycerolphosphate [R&D Systems]); (dexamethasone, ascorbate-phosphate, proline, pyruvate and TGF- β3 [R&D Systems]) and DMEM (Sigma) supplemented with 5% FBS (Innovative Research), 50 ng ml^-1 ^FGF-2 (R&D Systems), 5 ng ml^-1 ^IGF-1 (R&D Systems) and 20 ng ml^-1 ^of BMP-2 (R&D Systems) respectively. Differentiation media was refreshed every 3 days.

### Co-culture system

Neonatal ventricular cardiomyocytes were cultured for three days to subconfluency on glass cover slips placed in 6-well tissue culture treated plates (BD Biosicience). Third passage BM-MSCs were transduced overnight with the BacMam 2.0 GFP transduction control (Invitrogen), trypsinized with 0.25% Trypsin EDTA (Invitrogen), and layered over the subconfluent layer of ventricular cardiomyocytes at a density of 1 × 10^6 ^MSCs per plate to make up a co-culture system. The cells were incubated at 37°C in 5% CO_2_. Cell fusion was determined by dual expression of GFP (FITC) and cardiac specific proteins (TRITC) by GFP positive BM-MSCs in close proximity to NVCMs (TRITC only).

### Cell staining

Cells were fixed in 10% formaldehyde at room temperature for 5 minutes, and rinsed with isopropanol. Slides were incubated with Oil-red-O (Fisher) for 20 minutes at room temperature, and rinsed with distilled water. For Alizarin Red staining of mineral deposits, cells were fixed with 10% formaldehyde for 15 minutes, and rinsed with distilled water. Slides were incubated with Alizarin Red staining solution (Fisher) for 20 minutes at room temperature, and rinsed with distilled water. BM-MSCs were viewed under inverted phase-contrast microscope (BX61, Olympus; Tokyo, Japan).

### Immunofluorescence staining

For immunocytochemistry, cells were grown on glass cover slips (Fisher) to subconfluency, washed with PBS, fixed with 2% paraformaldehyde for 10 minutes at room temperature, quenched with 100 mM Glycine (pH7) for 5 minutes, permeabilized with 0.1% Triton X-100 for 5 minutes, blocked with 1% BSA in PBS for 20 minutes at room temperature, and sequentially incubated with respective primary antibodies at 4°C overnight and then with appropriate secondary antibodies for 1 hour at 37°C. Between steps, PBS was used for washes and cells were mounted with Prolong Gold Antifade with DAPI (Invitrogen). Confocal images of immunostained cells were obtained using a 40× oil objective on a Zeiss LSM 510 Laser Scanning Confocal Microscope (Zeiss, Frankfurt, Germany). Digitized confocal images were processed with Zeiss LSM Image Browser software, Rel. 4.2 and Adobe Photoshop.

### Assessment of telomerase activity

Telomerase activities from young and old rat MSCs were determined using the Quantitative Telomerase Detection Kit (MT3010; Allied Biotech, Inc., Germantown, MD) according to the manufacturer's protocol. Briefly, cell pellets were washed, resuspended in Lysis Buffer and incubated for 30 minutes on ice. Protein concentration was determined for each sample and appropriate volumes of samples, heat inactivated extracts and template controls were dispensed into individual PCR tubes containing a pre-made master mix supplied by the manufacturer. The Bio-Rad CFX96 Real-Time PCR Detection System (Bio-Rad, Hercules, CA USA) was used to collect the appropriate C_q _values for each sample.

### Real-time reverse transcription PCR (qRT-PCR)

Total RNA was extracted from rat BM-MSCs using Trizol Reagent (Invitrogen) according to the manufacturer's protocol. For qPCR, 2 ug of total RNA was reverse transcribed with the RT^2 ^First Strand Kit (Applied Biosystems, Foster City, CA) according to the manufacturer's protocol. qPCR was performed using the SsoFast EvaGreen supermix (Bio-Rad) according to the manufacturer's instructions. The cycling profile for real-time PCR (40 cycles) was as follows: 30 seconds at 95°C for enzyme activation, 5 seconds at 95°C for initial denaturation, 5 seconds at 65°C for annealing/extension and a 5 second melt curve step at 65-95°C. Gene analysis was performed using the Bio-Rad CFX Manager software (Bio-Rad). Gene expression is normalized relative to unstimulated cells and fold variation is GAPDH normalized. The primer sequences used have been shown in Additional file 4.

### Statistical Analysis

All differentiation and co-culture experiments were carried out in triplicate (n = 3) per animal in each age group (n = 4). qPCR experiments represent three biologically independent experiments realized in duplicate. Data are presented as mean ± SEM. Analyses of variance (ANOVA) were performed using Sigma Plot software from Systat Software, Inc (Chicago, IL, USA) followed by Tukey's multiple comparison tests to establish statistical significance between experimental groups at the p < .05 (*) level.

## List of Abbreviations

BM-MSC: bone marrow-derived mesenchymal stem cells; BMP-2: bone morphogenetic protein 2; BSA: bovine serum albumin; cTnI: cardiac troponin I; cTnT: cardiac troponins T; cTnC: cardiac troponin C: cTrpm: cardiac tropomyosin; DAPI: 4': 6-diamidino-2-phenylindole; DMEM-HG: Dulbecco's modified Eagle's medium--high glucose; EGF: epidermal growth factor; FACS: fluorescence-activated cell sorting; FBS: fetal bovine serum; FGF-2: fibroblast growth factor 2; FITC: fluorescein isothiocyanate; G-CSF: granulocyte colony stimulating factor; IGF-1: insulin-like growth factor 1; NVCM: neonatal ventricular cardiomyocytes; OBM-MSC: old bone marrow mesenchymal stem cells; PBS: phosphate buffered saline; PE: phocoerythrin; TRITC: tetramethylrhodamine isothiocyanate; VEGF: vascular endothelial growth factor; YBM-MSC: young bone marrow mesenchymal stem cells

## Authors' contributions

FZA and PBC conceived and designed the experiments. FZA conducted all experiments and data analysis. FZA and PBC wrote the article. All authors read and approved the final manuscript.

## Supplementary Material

Additional file 1 **MSC morphology and population size in young and old bone marrow: (A) Representative phase-contrast micrographs of resulting primary isolation BM-MSCs derived prior to the first passage at 24 hours (24 h), 48 hours (48 h), day 5 and day 7**. (B) Passage 0 BM-MSCs from individual young and old rats were trypsinized, and analyzed with a Cedex HiRes non-flow imaging cytometer to determine the average cell diameter of each individual MSC population. Representative phase-contrast micrographs from each experimental group are shown. Click here for file

Additional file 2 **(A) Columns show fluorescence signal intensity based on three independent differentiation experiments (n = 3) per animal**. The '‡' sign indicates a significant difference between the indicated group and any passage 3 groups from younger donors (*p <*0.05). Error bars designate means ± SEM (n = 4). Click here for file

Additional file 3 **(A) VEGF, EGF, IGF and G-CSF expression measured by qPCR**. (B) Nkx2.5, GATA 4, cTnC, cTnI, cTnT and cTrpm expression measured by qPCR following induction with growth factors and conditioned media. Results are normalized for expression in unstimulated cells at the same time of differentiation. Data represent mean ± SEM of three biologically independent experiments realized in duplicate. The '‡' sign indicates a significant difference between the indicated group and any passage 3 groups from younger donors (*p <*0.05). Click here for file

Additional file 4 **Primer sequences for specific genes Additional Statistical Analysis Fluorescence Signal Intensity Measurements**. Cardiomyogenic, osteogenic, chondrogenic and adipogenic differentiation was carried out on each individual cell line from each animal (n = 4, per group). Three independent differentiation experiments (n = 3) were carried out for each cell line. Zeiss LSM Image Browser software, Rel. 4.2 was used to determine percent fluorescence intensity. Differences between groups following immunocytochemical evaluation were compared by using 1-way ANOVA. Tukey's multiple comparison test was used to establish statistical significance between experimental groups at the p < .05 (*) level. Negative controls for all immunocytochemistry experiments represent secondary antibody staining only. Click here for file
