# Supplementary figures and images for: Age-related changes in rat bone-marrow mesenchymal stem cell plasticity
Source: BMC Cell Biol. 2011 Oct 12;12:44. doi: 10.1186/1471-2121-12-44 (PMC3204286; doi:10.1186/1471-2121-12-44)

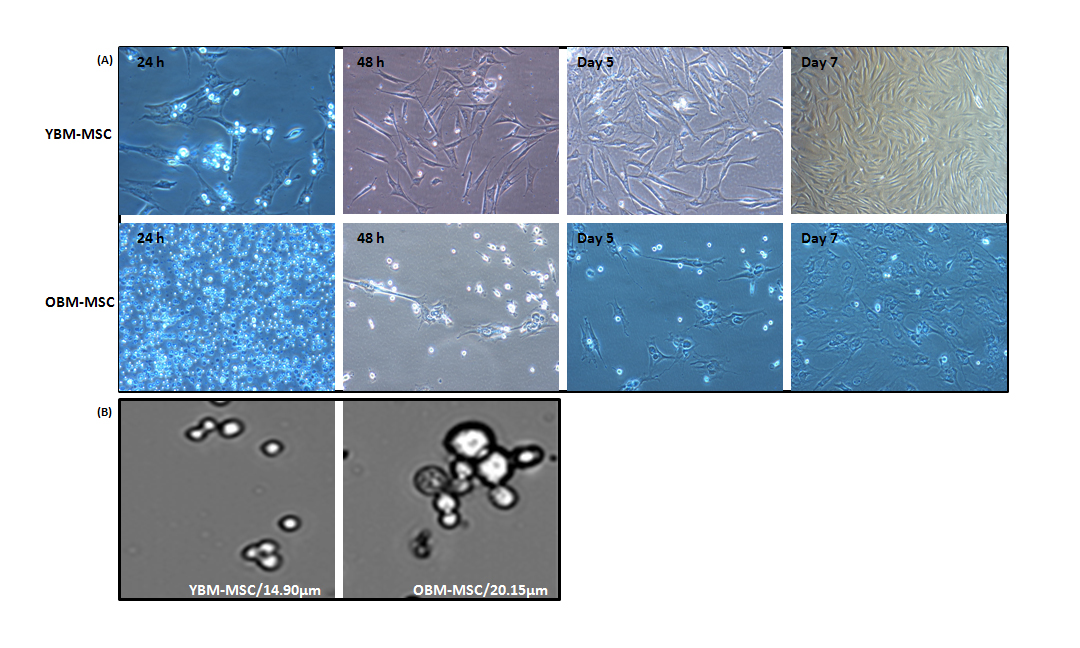

Supplement: Additional file 1 — MSC morphology and population size in young and old bone marrow: (A) Representative phase-contrast micrographs of resulting primary isolation BM-MSCs derived prior to the first passage at 24 hours (24 h), 48 hours (48 h), day 5 and day 7. (B) Passage 0 BM-MSCs from individual young and old rats were trypsinized, and analyzed with a Cedex HiRes non-flow imaging cytometer to determine the average cell diameter of each individual MSC population. Representative phase-contrast micrographs from each experimental group are shown. [file 1471-2121-12-44-S1.JPEG]

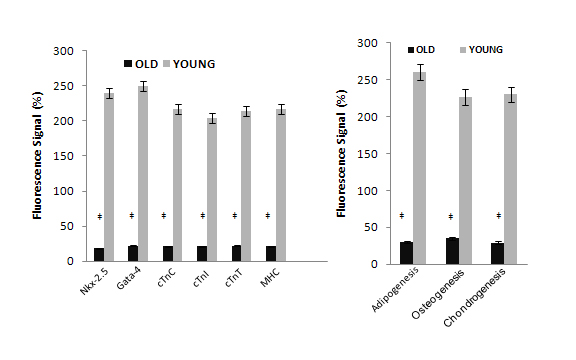

Supplement: Additional file 2 — (A) Columns show fluorescence signal intensity based on three independent differentiation experiments (n = 3) per animal. The '‡' sign indicates a significant difference between the indicated group and any passage 3 groups from younger donors (p <0.05). Error bars designate means ± SEM (n = 4). [file 1471-2121-12-44-S2.JPEG]

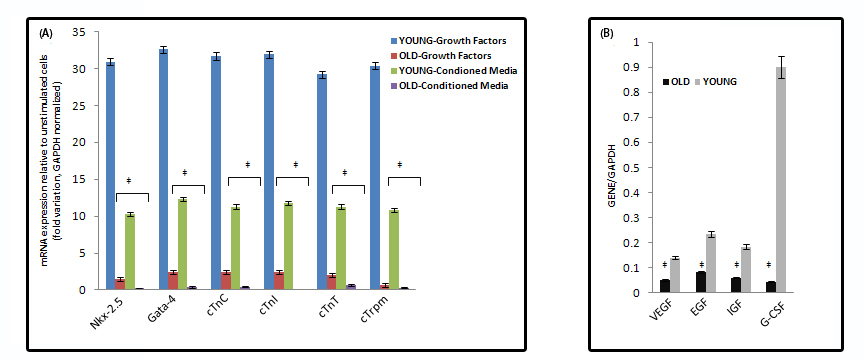

Supplement: Additional file 3 — (A) VEGF, EGF, IGF and G-CSF expression measured by qPCR. (B) Nkx2.5, GATA 4, cTnC, cTnI, cTnT and cTrpm expression measured by qPCR following induction with growth factors and conditioned media. Results are normalized for expression in unstimulated cells at the same time of differentiation. Data represent mean ± SEM of three biologically independent experiments realized in duplicate. The '‡' sign indicates a significant difference between the indicated group and any passage 3 groups from younger donors (p <0.05). [file 1471-2121-12-44-S3.JPEG]
