# Supplementary material for: Age-related changes in rat bone-marrow mesenchymal stem cell plasticity
Source: BMC Cell Biol. 2011 Oct 12;12:44. doi: 10.1186/1471-2121-12-44 (PMC3204286; doi:10.1186/1471-2121-12-44)
Supplement: Additional file 4 — Primer sequences for specific genes Additional Statistical Analysis Fluorescence Signal Intensity Measurements. Cardiomyogenic, osteogenic, chondrogenic and adipogenic differentiation was carried out on each individual cell line from each animal (n = 4, per group). Three independent differentiation experiments (n = 3) were carried out for each cell line. Zeiss LSM Image Browser software, Rel. 4.2 was used to determine percent fluorescence intensity. Differences between groups following immunocytochemical evaluation were compared by using 1-way ANOVA. Tukey's multiple comparison test was used to establish statistical significance between experimental groups at the p < .05 (*) level. Negative controls for all immunocytochemistry experiments represent secondary antibody staining only. [file 1471-2121-12-44-S4.DOC]

| Gene | Primer Sequence | Product size (bp) |
| --- | --- | --- |
| GAPDH  Oct-4  Sox-2  NANOG  VEGF  IGF  EGF  G-CSF  GATA 4  Nkx2.5  cTnC  cTnI  cTnT  cTropomyosin | 5’-AACTTTGGCATCGTGGAAGGG-3’  5’-AGGGATGATGTTCTGGGCTGC-3’    5’-AGAACCGTGTGAGGTGGA AC-3’  5’-GCC GGT TACAGAACCAGACT-3’  5’-GCAGTACAACTCCATGACCAGC-3’  5’-GCTGGTCATGGAGTTGTACTGC-3’    5’-TCTCTCTACCATTCTGAGCCTGAGC-3’  5’-GCCGTTGCTAGTCTTCAACCATTGG-3’  5’-CTGCCTGGAAGAATCAGGAG-3’  5’-GAGGAGGAGGAGCCATTACC-3’  5’-GCTGCTGAAGCCGTTCATTTA-3’  5’-AAGAAAGGGCAGGGCTAATGG-3’  5’-ACAACACCCTGGTCTGGAAG-3’  5’-GCCCTTCTGGTTGTTGACAT-3’  5’- CCTTGGAGCAAGTGAGGAAG-3’  5’-TTGGGGATACCCAGAGAGTG-3’  5’-TATTTTGAGCGAGTTGGGCCT-3’  5’-TCGGTGTGCTCCCCTTTATTT-3’  5’-GGATTTCACACCCACACTTGC-3’  5’-TCCGGGTCCTGATATGGAATC-3’  5’-**CAGCAAAGGGAAGTCTGAGG-3’**  5’-**CTTCCGTGATGGTCTCACCT-3’**  5’-CGTGTGGACAAAGTGGATGAA-3’  5’-AGAGTGGGCCGCTTAAACTTG-3’  5’-GGACAAGGCAGAAGAGGTTG-3’  5’-ACCAAGTTGGGCATGAAGAG-3’  5’-AAAGTATGAAGAGGTGGCCCG-3’  5’-GCAGCCATTAATGCTTTCAAGG-3’ | 130  125  128  135  119  141  145  130  146  135  128  125  127  122 |
